# Gram Scale Synthesis of Dual-Responsive Dendritic Polyglycerol Sulfate as Drug Delivery System

**DOI:** 10.3390/polym13060982

**Published:** 2021-03-23

**Authors:** Felix Reisbeck, Alexander Ozimkovski, Mariam Cherri, Mathias Dimde, Elisa Quaas, Ehsan Mohammadifar, Katharina Achazi, Rainer Haag

**Affiliations:** Institute of Chemistry and Biochemistry, Freie Universität Berlin, Takustr. 3, 14195 Berlin, Germany; freisbeck@zedat.fu-berlin.de (F.R.); saschaoz@zedat.fu-berlin.de (A.O.); mcherri6@zedat.fu-berlin.de (M.C.); mathias.dimde@fu-berlin.de (M.D.); equaas@zedat.fu-berlin.de (E.Q.)

**Keywords:** drug delivery system, dual-responsiveness, dendritic polyglycerol sulfates

## Abstract

Biocompatible polymers with the ability to load and release a cargo at the site of action in a smart response to stimuli have attracted great attention in the field of drug delivery and cancer therapy. In this work, we synthesize a dual-responsive dendritic polyglycerol sulfate (DR-dPGS) drug delivery system by copolymerization of glycidol, ε-caprolactone and an epoxide monomer bearing a disulfide bond (SSG), followed by sulfation of terminal hydroxyl groups of the copolymer. The effect of different catalysts, including Lewis acids and organic bases, on the molecular weight, monomer content and polymer structure was investigated. The degradation of the polymer backbone was proven in presence of reducing agents and *candida antarctica* Lipase B (CALB) enzyme, which results in the cleavage of the disulfides and ester bonds, respectively. The hydrophobic anticancer drug Doxorubicin (DOX) was loaded in the polymer and the kinetic assessment showed an enhanced drug release with glutathione (GSH) or CALB as compared to controls and a synergistic effect of a combination of both stimuli. Cell uptake was studied by using confocal laser scanning microscopy with HeLa cells and showed the uptake of the Dox-loaded carriers and the release of the drug into the nucleus. Cytotoxicity tests with three different cancer cell lines showed good tolerability of the polymers of as high concentrations as 1 mg mL^−1^_,_ while cancer cell growth was efficiently inhibited by DR-dPGS@Dox.

## 1. Introduction

The development of novel drug delivery systems (DDS) for application in chemo-therapy has been widely studied. A major drawback of established chemotherapeutic agents such as Doxorubicin (DOX) is their poor solubility in water and unspecific toxicity due to lack of targeting [1]. One method to overcome these drawbacks is the use of polymeric DDS for the encapsulation and targeted release of the drug at the cancer site. Prominent examples for the basis of DDS include natural polymers such as chitosan and hyaluronic acid (HA) and synthetic polymers such as polyglutamic acid (PGA), poly(lactic-co-glycolic acid) (PLGA), polyethylene glycol (PEG) and dendritic polymers [2,3,4,5]. The utilization of DDS allows for prolonging circulation time of drug conjugates and therefore, enhanced permeation and retention can be achieved as passive targeting due to the leaky vasculature of tumors. However, in recent years research revealed that this effect displays heterogeneity depending on the tumor and that the uptake of nanoparticles in cancerous tissue is due to an active mechanism rather than passive accumulation [6,7].

In order to enable site-specific release of the drug, inherent properties of cancerous tissue can be exploited. Stimuli such as the decreased pH regime, overexpression of enzymes, presence of reactive oxygen species (ROS) as well as an increased redox potential due to the elevated concentration of the tripeptide glutathione (GSH) are possible rationales for the design of DDS and the incorporation of stimuli-responsive motifs into the polymer structures [8].

Dendritic polyglycerol sulfate (dPGS) has been vastly investigated in the past years as a potential candidate for a variety of medical applications [9]. It exhibits features, such as well-established synthesis, tunable molecular weight, size, surface charge, and flexible globular shape. Furthermore, the polyether backbone as well as its overall negative surface charge lead to biocompatibility up to high concentrations and high water solubility, while maintaining an inertness of the polymer to biological components such as proteins or phospholipid membranes [10]. Originally developed as an alternative for heparin [11], in vivo experiments showed a very high anti-inflammatory activity of dPGS [12]. This led to the investigation of dPGS as potential candidate in different inflammation-related applications, including bone targeting and treatment of osteoarthritis and neurological disorders [13,14,15]. As inflammation (and thus a higher L/P-selectin concentration) is concomitant with tumor growth, dPGS-based systems exhibited an enhanced tumor targetability, which could be shown by in vivo experiments [16,17,18].

Taking all these advantages into account, dPGS would be a potential candidate for the development of a drug delivery system. However, one drawback for such applications is its extreme hydrophilicity, hampering the encapsulation of (mostly hydrophobic) anticancer drugs as well as lack of biodegradability in physiological medium. Thus far, several different works have attempted to improve these drawbacks by incorporating hydrophobic and cleavable segments in the dPG backbone [19,20,21,22]. However, these attempts have always been challenging due to the laborious procedures and low synthesis scale.

**Figure 1:**
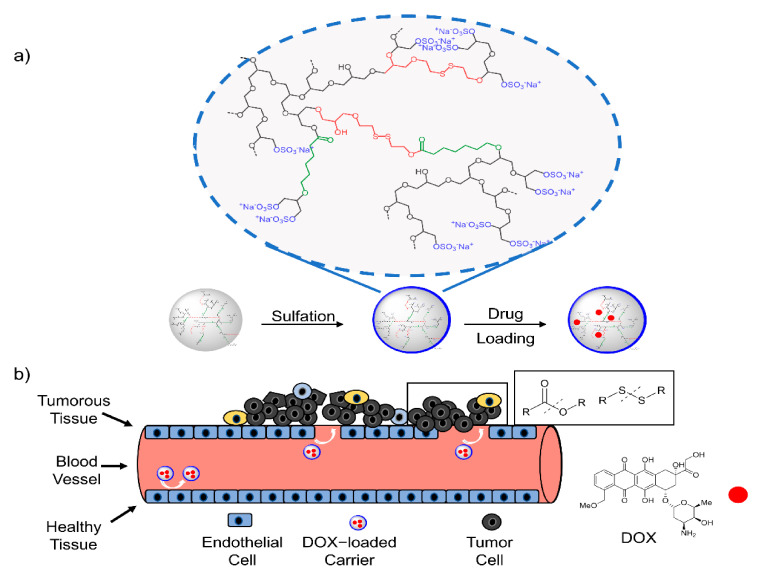
Schematic illustration of synthesis and DOX-loading of the polymeric DDS with subsequent drug release. (**a**) Synthesis of copolymer, sulfation and Dox-loading; (**b**) tumor targeting and underlying mechanism of drug release.

In this work, we present a new method for gram scale synthesis of a DDS based on a sulfated dendritic terpolymer of glycidol, ε-caprolactone and 2-((2-(oxiran-2-ylmethoxy)ethyl)disulfanyl)ethan-1-ol (SSG) for the potential targeted delivery of Doxorubicin (Figure 1). First, the optimal reaction conditions with different catalysts for the copolymerization were screened. The optimized copolymer was then further sulfated and loaded with the hydrophobic anticancer drug Doxorubicin (DOX). As noted, the sulfation of these carriers leads to an increased tumor targetability, whereas the incorporation of ester groups and disulfide bonds allows for the degradation and drug release at the tumor site. Although numerous publications focusing on the controlled and targeted drug delivery of anticancer drugs have been reported, developing new methods for the synthesis of low-cost biocompatible drug delivery systems in gram scale as well as scalable methods for drug loading are highly demanded for further biomedical applications. This novel DDS can be synthesized in gram scale within two facile reaction steps, shows high tumor targeting and its degradation under enzymatic and/or reductive conditions renders the drug release of DOX into cells.

## 2. Materials and Methods

Potassium tert-butoxide was purchased from ABCR (Karlsruhe, Germany). Anhydrous Toluene and DTT were bought from Acros (Geel, Belgium). Diphenyl phosphate was provided by TCI Chemicals (Tokyo, Japan). 2-Hydroxyethyl disulfide, DCM, Sn(Oct)_2_, Strontium ispopropoxide, Mg(HMDS)_2_, TBD, DBU, TCEP, and GSH were purchased from Sigma Aldrich (Merck KGaA, Darmstadt, Germany) and used without further purification. *Tert*-Butanol was bought from Grüssing GmbH (Filsum, Germany). Glycidol (Acros) and ε-CL (TCI) were distilled prior to use and stored in a Schlenk flask over a molecular sieve (4 Å). Cyanine-5-amine was purchased from Lumiprobe (Hannover, Germany). Pur-A-Lyzer Maxi 6000 Dialysis Kit was bought from Sigma Aldrich. Doxorubicin hydrochloride was purchased from ABCR GmbH.

### 2.1. Instrumentation

NMR spectra were recorded on a Jeol Eclipse 500 MHz (Tokyo, Japan) or a Bruker AVANCE III 700 MHz spectrometer (Billerica, MA, USA). Chemical shifts δ were reported in ppm and the deuterated solvent peak was used as internal standard. All spectra were recorded at 300 K. NMR data were reported including: chemical shift, multiplicity (s = singlet, d = doublet, t = triplet, m = multiplet), integration and coupling constants (s) in Hertz (Hz). Multiplets were recorded over the range (ppm) in which they appear in the spectrum.

Elemental analysis was performed with a VARIO EL III (Elementar).

GPC measurements in DMF were performed with on a customized chromatography system (PSS Polymer Standards GmbH, Mainz, Germany). A 5 cm precolumn (PSS-SDV in DMF, 5 µm particle size) coupled with a 30 cm column (PSS SDV linear M in DMF, 5 µm particle size) and a differential refractometer was used to separate and analyze the samples. The mobile phase was DMF (10 mM LiBr) at a flow rate of 1.0 mL min^−1^. The columns were heated at 50 °C, while the differential refractometer detector was kept at 35 °C. For each measurement, 50 µL of a prefiltered (PTFE 0.2 µm) 1.5 mg mL^−1^ sample solution was injected. The data were processed using the WinGPC unichrome software from PSS. Molecular weights and molecular weight distribution were obtained relative to a poly(methyl methacrylate) standard.

For the purification of the copolymer, tangential flow filtration was performed using a 10 kDa regenerated cellulose cassette (Merck) in a cassette holder (Sartorius). The flow of the solution through the system was induced by a peristaltic pump (Gibson). The rotor speed was kept at the maximal operating one. The sulfated copolymers were purified by dialysis in brine to water. Benzoylated cellulose membrane (Sigma Aldrich, MWCO = 2 kDa) was chosen for this step.

UV/Vis measurements were conducted on an Agilent Cary 8454 UV-visible spectrophotometer, using half-micro quartz cuvettes.

Fourier transform infrared spectroscopy (FTIR) spectra were recorded on a Nicolet AVATAR 320 FT-IR 5 SXC (Thermo Fisher Scientific, Waltham, MA, USA) with a DTGS detector from 4000 to 650 cm^−1^. Sample measurement was performed by dropping a solution of the compound and letting the solvent evaporate for a few seconds.

Raman spectra were recorded on a Bruker (Karlsruhe, Germany) MultiRAM II equipped with a low-temperature Ge detector (1064 nm, 100–180 mW) with 256 scans at a resolution of 2 cm^−1^.

### 2.2. Synthesis

#### 2.2.1. 2-((2-(oxiran-2-ylmethoxy)ethyl)disulfanyl)ethan-1-ol (SSG Monomer)

SSG Monomer was synthesized according to a modified published procedure [23]. Briefly, a solution of 2-Hyroxyethyl disulfide (13.9 g; 90 mmol; 1.0 equiv.) in *tert*-Butanol (150 mL) was added dropwise via dropping funnel to a solution of KO^t^Bu (10.1 g; 90 mmol; 1.0 equiv.) in *tert*-Butanol (225 mL). After 4 h, excess epichlorohydrin (64.4 mL; 600 mmol; 6.7 equiv.) was added dropwise and the reaction mixture was stirred overnight at room temperature. The formed salt was filtered off and the solvent removed under reduced pressure. The crude product was dissolved in DCM (150 mL), extracted with water (3 × 50 mL) and dried over Na_2_SO_4_. After purification by column chromatography (eluent EtOAc 4: 1 Hexane *v*/*v*), the product (5.9 g; 28 mmol; 31%) was obtained as a yellow oil. ^1^H NMR spectra were in accordance with reported data. The successful synthesis of the monomer was confirmed by ^1^H and ^13^C NMR spectroscopy (see Supplementary Information).

#### 2.2.2. Polymerization Procedure

The SSG monomer (3.15 g, 15 mmol, 1.0 equiv.), glycidol (10 mL; 150 mmol; 10 equiv.) and ε-CL (1.66 mL; 15 mmol; 1.0 equiv.) were mixed and formed a clear solution. The catalyst (1.5 mmol; 0.1 equiv.) was dissolved in anhydrous toluene (7.5 mL) and divided into 5 equal portions. To initiate the reaction, the monomer solution (1.0 mL) and the catalyst solution (2.5 mL) were added to a 2-necked flask with installed mechanical stirrer at 70 °C with a stirring speed of 120 rpm. The remaining monomer solution was added via a syringe pump over the next 8 h, whereas a portion of the catalyst solution was added to the reaction mixture every 2 h. The reaction was kept at 70 °C under constant stirring for 48 h and terminated by the addition of water (30 mL). Crude products were purified by tangential flow filtration (MWCO 10 kDa), dried via lyophilization, and analyzed by means of GPC, NMR, IR and Raman spectroscopy as well as elemental analysis. For Sn(Oct)_2_ and DBU catalysts, no toluene was used as solvent, since these are liquids in pure form.

#### 2.2.3. Sulfation of Polymers

This reaction was performed according to an established protocol [24]. The unsulfated polymer was dried at 60 °C under high vacuum overnight and then dissolved in anhydrous DMF (concentration 0.029 g/mL). Then, a solution of sulfur trioxide pyridine complex (1.5 eq/OH group) in anhydrous DMF (concentration 0.1 g/mL) was added via syringe pump over 2 h and the reaction was kept at 60 °C for 24 h. The pH was adjusted to 7 with 1 M NaOH, solvent was removed under reduced pressure and the polymer dissolved in brine. Finally, dialysis (MWCO 2 kDa) was performed with brine solution, slowly decreasing the salt content to pure water over 96 h. After lyophilization, the product was obtained as a white to yellowish solid. The products were analyzed by means of NMR, GPC and IR spectroscopy. The degree of sulfation was calculated based on elemental analysis.

#### 2.2.4. Preparation of Doxorubicin (Free Base)

DOX·HCl (100 mg; 0.172 mmol, 1.0 equiv.) was dissolved in water (200 mL) and added with DCM (200 mL) to a separation funnel. Then, NEt_3_ (1 mL; 7.2 mmol; 42.0 equiv.) was added, the organic layer was separated, and the aqueous phase extracted with DCM (5 × 50 mL). The organic phase was dried over Na_2_SO_4_, the solvent removed under reduced pressure and the product dried under high vacuum. The product was obtained in quantitative yield and stored in the freezer. ESI-MS: *m*/*z*. Calculated 544.1819 g/mol. Found 544.1908 g/mol ([M+H]+).

### 2.3. Drug Encapsulation

To 10 mg of Doxorubicin (free base), 1 mL of a solution of the sulfated polymer in Milli-Q water (100 mg/mL) was added dropwise at room temperature at 1200 rpm. In order to separate conjugates from the free drug, the solution was transferred into a Falcon tube, centrifuged at 4000 rpm for 5 min and finally purified by column chromatography with a Sephadex G-25. Conjugates were partially lyophilized and the drug-loading content was determined by UV/Vis measurements.

### 2.4. Degradation Study with Reducing Agents

For degradation studies, 10 mM solutions of DTT, TCEP and GSH were prepared. For the latter, the pH was adjusted to 7.4 with 1 M NaOH. Solutions were degassed meticulously for 2 h. Polymers (40 mg) were dissolved in the respective solutions (10 mg mL^−1^) and incubated at 37 °C for 24 h. The solutions were dialyzed (MWCO 1 kDa) against water and the dialysate was replaced every 6 h. Finally, solutions were lyophilized and analyzed by means of ^1^H NMR. As a control, polymer solutions were prepared in PBS and processed in the same manner.

### 2.5. Degradation Study with Lipase B

This degradation study was performed according to an established protocol [25]. Briefly, polymers (30 mg) were dissolved in PBS (60 mL). Then, *Candida antarctica* Lipase B (60 mg; 200 wt% of polymer) and a few drops of n-Butanol were added and incubated at 37 °C for 72 h. The resin was filtered off, the solution lyophilized and analyzed by means of ^1^H NMR. As a control, the same solution was prepared and processed without the addition of resin-immobilized enzyme.

### 2.6. Release Study of Doxorubicin

DR-dPGS@DOX conjugates with an overall DOX content of 0.2 mg were dissolved in solutions (2 mL) of the reducing agents and/or CALB resin. Prior to use, all solutions were treated exactly as for the degradation study. As controls, DR-dPGS@DOX in PBS without any reducing agent and pure DOX in PBS were used. All solutions were transferred into a Float-a-lyzer dialysis kit (MW 6–8 kDa) inside a Falcon tube filled with 30 mL of the respective medium and incubated at 37 °C. The DO content inside the dialysis kit was measured at constant time points (0 h, 0.5 h, 1 h, 2 h, 3 h, 4 h, 5 h, 6 h, 8 h, 24 h, 26 h, 30 h, 48 h, 54 h, and 72 h) in order to measure the released drug. For normalization, the initial absorbance at 0 h was set to 100% content and the cumulative released was calculated based on that.

### 2.7. Cytotoxicity Studies

The effect of the compounds on three cancer cell lines, A549, HeLa and MCF-7, was determined using the cell viability assay Cell Counting Kit 8 (CCK-8) from Sigma Aldrich Chemie GmbH (Taufkirchen, Germany) according to the manufacturer’s instructions. A549 (DSMZ no.: ACC 107), HeLa (DSMZ no.: ACC 57) and MCF-7 (DSMZ no.: ACC 115) cells were obtained from Leibniz-Institute DSMZ—Deutsche Sammlung von Mikroorganismen und Zellkulturen GmbH and cultured in Dulbecco’s Modified Eagle’s Medium (DMEM) supplemented with 10% (*v*/*v*) FBS, 100 U/mL penicillin and 100 µg/mL Streptomycin (all from Gibco BRL, Eggenstein, Germany). Cells were regularly subcultured at least twice a week when they reached 70% to 90% confluency. For the cytotoxicity assay, 90 µL of a cell suspension in DMEM containing 5 × 104 cells per mL were seeded in each inner well of a 96-well plate and incubated over night at 37 °C and 5% CO2. In the outer wells, 90 µL DMEM without cells were added. The next day, serial dilutions of all the samples were prepared and10 µL each were added to the cells in triplicates and in addition to one outer well for background correction. SDS (1%) and nontreated cells served as controls. After another 48 h at 37 °C and 5% CO2, the CCK-8 solution was added (10 µL/well) and absorbance at a measurement wavelength of 450 nm and a reference wavelength of 650 nm was measured after approximately 3 h incubation using a Tecan plate reader (infinite pro200, TECAN-reader Tecan Group Ltd. Männedorf, Switzerland). Measurements were performed in triplicate. The cell viability was calculated by setting the nontreated control to 100% after subtracting the background using Excel software. All cell experiments were conducted according to German genetic engineering laws and German biosafety guidelines in the laboratory (safety level 1).

IC50 values were calculated with GraphPad Prism 6.01 using the log(inhibitor) vs. normalized response equation.

### 2.8. Confocal Laser Scanning Microscopy (CLSM)

The uptake of Cy5-labeled DR-dPGS and DR-dPGS@DOX in HeLa cells was analyzed using Confocal Laser Scanning Microscopy (CLSM). The cells were propagated as described above. For CLSM, HeLa cells were seeded in 8-well ibidi slides (ibidi treat) in 270 µL DMEM. After cell attachment 4 h to 24 h, a postseeding 30 µL of solution containing compound were added for 3 h or 20 h. Before imaging, the cells were stained with Hoechst 33342 (1 µg/mL), washed with PBS and covered with fresh cell culture medium (DMEM). Confocal images were taken with an inverted confocal laser scanning microscope Leica DMI6000CSB SP8 (Leica, Wetzlar, Germany) with a 63x/1.4 HC PL APO CS2 oil immersion objective using the manufacture given LAS X software in sequential mode with the following channel settings: Transmission Ch (grey intensity values), excitation laser line 405 nm, detection of transmitted light (photomultiplier); Ch1 (Hoechst 33342): excitation laser line 405 nm, detection range 410 nm–485 nm (hybrid detector); Ch2 (Doxorubicin): excitation laser line 488 nm, detection range 496 nm–629 nm (hybrid detector); Ch3 (Cy5 dye): excitation laser line 488 nm, detection range 638 nm–797 nm (photomultiplier).

## 3. Results

### 3.1. Polymer Synthesis and Analysis

All polymers were synthesized by ring-opening copolymerization (ROP) of glycidol, ε-caprolactone (ε-CL) and 2-((2-(oxiran-2-ylmethoxy)ethyl)disulfanyl)ethan-1-ol (SSG), using Lewis acids and organic bases as catalysts in a one-pot gram-scale reaction. Due to the fact that a higher PCL content can lead to water solubility issues, we decided to keep the ε-CL content fixed at 10 mol% in the feed. We sought the optimal conditions for a gram-scale batch size polymerization at 70 °C with a catalyst that renders reasonable yields, molecular weights above 10 kDa, low polydispersity (Đ), a sufficient degree of branching (DB) and incorporation of intact ester structures of the CL and disulfide bonds of the SSG, respectively. As has been stated by Kizhakkedathu et al. before, typical temperatures for the ring-opening polymerization (ROP) of such monomers is around 95 °C; however, the SSG monomer is not stable and disulfide linkages undergo decomposition into thiols and thioethers [26]. Similar observations were made, and therefore, the polymerization temperature was kept strictly at 70 °C.

In order to find optimal conditions for the synthesis of the copolymers, a wide scope of catalysts were screened. Thus far, a variety of catalysts has been studied for the ROP of caprolactones and oxiranes including Lewis acids such as Sn(oct)_2_, strontium isporopoxide (Sr(OiPR)_2_), Mg(HMDS)_2_ and organocatalysts such as 1,8-diaza-[5.4.0]undec-7-ene (DBU) and 1,5,7-triazabicyclo[4.4.0]dec-5-ene (TBD) and diphenyl phosphate (DPP) [27,28,29]. In our group, the synthesis of dPG-PCL copolymers was reported recently, utilizing Sn(oct)_2_ as a suitable catalyst [30]. All aforementioned catalysts were used for ROP of glycidol (Gly), ε-CL and SSG with the feed molar ratio of [Gly]/[ε-CL]/[SSG]:[10]/[1]/[1] (Table 1).

**Figure 2:**
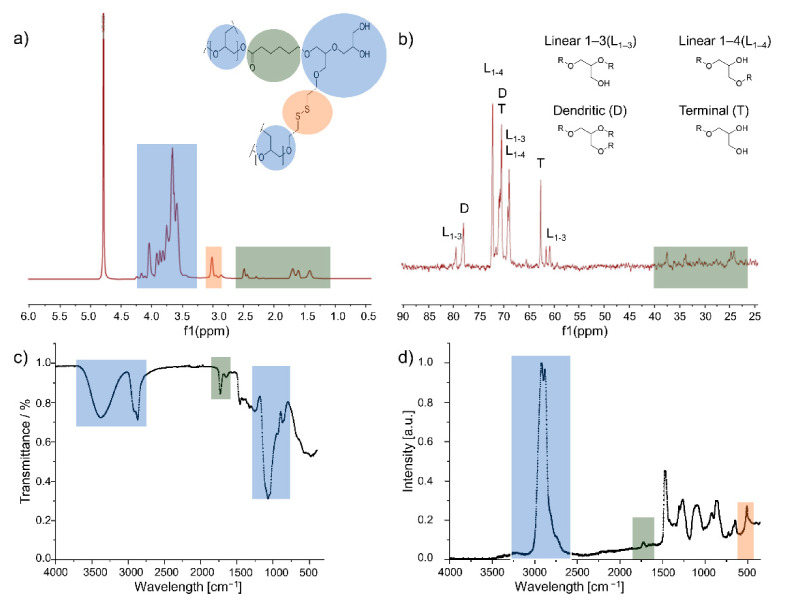
Analysis of the synthesized copolymers with color-coded structural units of glycidol (blue), SSG (red) and ε-CL (grey). (**a**) ^1^H NMR spectrum with assignment of specific peaks of the three different monomer species; (**b**) inverse gated ^13^C NMR spectrum with assignment of the signals of linear, dendritic and terminal carbon signals for the calculation of the degree of branching; (**c**) IR spectrum; (**d**) Raman spectrum.

All polymers were analyzed by means of ^1^H and ^13^C NMR, GPC, IR and Raman spectroscopy. Typical spectra of products can be seen in Figure 2 for the example of the Sr(OiPr)_2_-catalyzed polymer.

As the ^1^H NMR spectrum (Figure 2a) displays quantifiable signals of the dPG backbone (4.2–3.5 ppm), the signals of the protons of the methylene groups next to the disulfide bonds (3.0 ppm) and the aliphatic units of ε-CL, particularly the methylene bridge next to the carbonyl group (2.5 ppm), it is the proof of the incorporation of the three monomers into the polymer backbone and the basis for the determination of disulfide and ε-CL content in Table 1. The monomer content was calculated based on NMR and elemental analysis (Table 1). The details of the calculations are explained in the Supplementary Information. As opposed to the tedious synthesis of perfect dendrimers, DR-dPGS can be synthesized in a large batch size within two reaction steps and maintain the advantages of its dendrimer analogue; however, the degree of branching is only 56% (Table 1).

Besides the composition of the polymers, their structure is of high importance for the application as a DDS. Therefore, the degree of branching was calculated based on the definition by Frey [31], by using integrals obtained by overnight IG ^13^C NMR spectra:

(1) $$DB=\;\frac{2\;D}{2\;D\;+\;L}$$

where *D* is the relative integral of dendritic units and *L* is the relative integral of linear units of type L_1–3_ and L_1–4_ as indicated in Figure 2b.

Furthermore, IR spectroscopy (Figure 2c) was used to show the presence of the structural units in the copolymer. The broad peaks around 3400 cm^−1^ and 3000 cm^−1^ can be assigned to the hydroxy groups of the polyether and its C-H bonds, respectively. The presence of ε-CL can be determined with this method due to the strong carbonyl bond in IR spectroscopy at 1725 cm^−1^. Moreover, the signal induced by the C-O bonds is strongly present at 1100 cm^−1^. As disulfide bonds cannot be detected easily with IR spectroscopy, Raman spectroscopy (Figure 2d) is a useful method to determine their presence and generate a more complete image of the polymer structure. The absorbance band at 500 cm^−1^ in the Raman spectrum is assigned to the disulfide bond of the SSG monomer [32]. The results lead to the conclusion that all monomers were incorporated into the polymer structure.

As noted, ideally catalysts render polymers with molecular weights above 10 kDa and narrow molecular weight distribution in good yields, a sufficient degree of branching (DB) and the incorporation of intact ester and disulfide bonds in the polymer. Taking the results from Table 1 into account, considering molecular weight, degree of branching, monomer content and potential catalyst toxicity, the copolymer synthesized with Sr(OiPr)_2_ was selected for further experiments and will be further referred to as Dual-Responsive dendritic Polyglycerol Sulfate DR-dPGS.

### 3.2. Degradation Study

**Figure 3:**
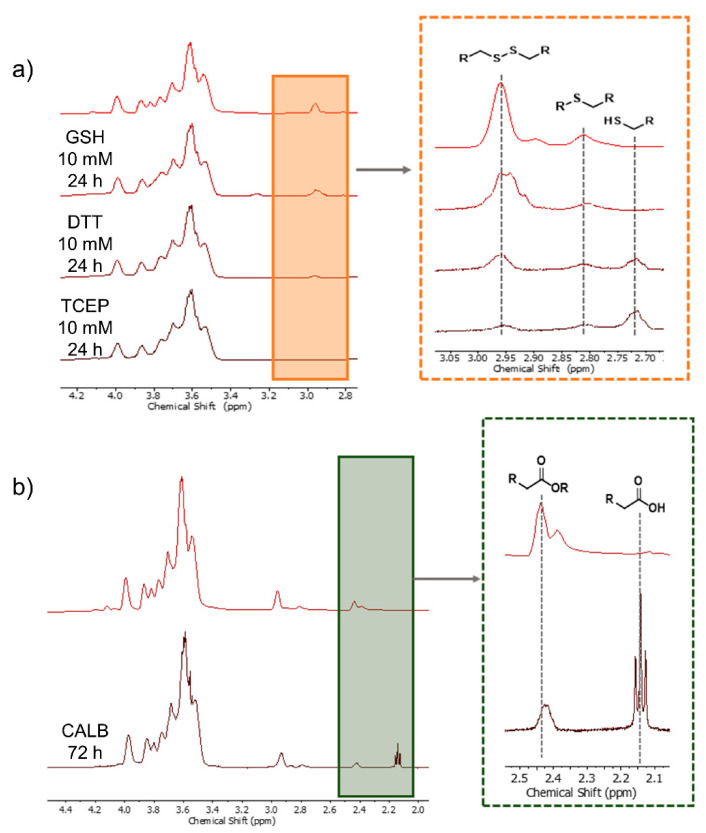
Degradation Study of DR-dPG. (**a**) ^1^H NMR spectra after 24 h with incubation at reductive conditions with GSH, DTT, and TCEP. The decrease in the integral of methylene group next to the disulfide and increase in its thiol counterpart are highlighted; (**b**) degradation under enzymatic conditions with CALB. The decrease in the methylene bridge in α-position to the ester and increase in its respective acid counterpart are highlighted.

In order to prove the dual-responsiveness of the system, it is necessary to show its degradation when being exposed to a reducing agent or an ester-cleaving enzyme. For this a degradation study using the reducing agents DTT, TCEP and the physiologically relevant GSH as well as the enzyme *Candida antarctica* Lipase B (CALB, commercially known as Novozyme-435) were used. Degradation under reductive and enzymatic conditions was monitored for 24 h and 72 h by using NMR spectroscopy (Figure 3).

The degree of degradation was deduced from the comparison of the relevant signals to the integral at 4.0 ppm, which stays constant during the entire process (Figure S1). As can be seen, the incorporated disulfides are cleaved as the integral decreases, whereas the thioether-related integral at 2.80 ppm and the thiol-related integral at 2.70 ppm increase (Figure 3a). As proof of principle, the reducing agents DTT and TCEP show a decrease in disulfide content of 79% and 92%, respectively. The physiologically more relevant GSH reduces the disulfide content in the copolymer of 31% within 24 h. Even though it is not as efficient as the other reducing agents, the ratio of GSH to its dimer GSSG is kept constant by enzymes in the cells and therefore is renewed more frequently than in a degradation study [33]. The incubation of the copolymer with CALB leads to a decrease in the α-carbonyl signal and an increase in its counterpart next to the carboxyl group (Figure 3b) and shows 74% ester degradation within 72 h.

### 3.3. Sulfation

DR-dPG was then further sulfated by an established procedure, using sulfur trioxide pyridine complex in DMF at 60 °C overnight [24]. The reaction was followed by dialysis against brine, slowly decreasing the salt concentration to pure water. The sulfated copolymer was then analyzed by means of zeta potential measurements and elemental analysis (EA). The latter is the basis for the calculation of the degree of sulfation (DS). The DS was calculated based on the results obtained in Table 1. A detailed calculation is given in the Supplementary Information.

For dPG, sulfation leads to roughly an increase of double the molecular weight, as statistically each glycidol unit leads to the formation of one hydroxyl group which can be converted into a sulfate group. Here, the Mn for DR-dPGS was calculated as 45.5 kDa (a detailed calculation is given in the Supplementary Information).

### 3.4. Drug-Loading and Release Study

**Figure 4:**
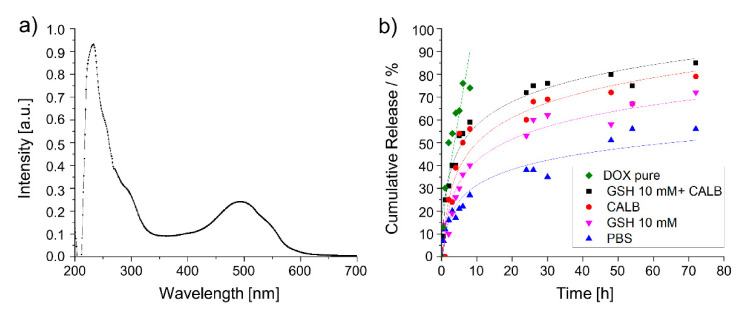
Determination and of drug loading content and subsequent release of DOX under reductive and/or enzymatic conditions. (**a**) UV/Vis spectrum of DR-dPGS@DOX; (**b**) release curve of DOX (linear fit), GSH+CALB, CALB, GSH, and PBS (Logarithmic Log3P1 fit).

Moreover, the efficacy of DR-dPGS in encapsulating Doxorubicin (free base) was investigated. For this purpose, the polymer was loaded with a targeted amount of 10 wt% and purified prior to analysis by UV/Vis measurements (Figure 4a).

The amount of loaded drug was calculated based on a calibration curve measured at different concentrations (Figure S2). The drug-loading capacity (DLC) was determined according to the following equation:

(2) $$DLC=\;\frac{m\;\left(Drug\right)}{m\;\left(total\right)-m\;\left(Drug\right)}\times 100$$

As successful loading of Doxorubicin and the degradation of the copolymer could be proven (Figure 4a), we next developed a set-up to monitor the triggered release of DOX under reductive or enzymatic degradation. As GSH concentrations of up to 10 mM can be reached in the cytosol, [34] we chose this for proof-of-concept experiments for the reductive environment. DOX release under enzymatic conditions was screened accordingly to the degradation study. Furthermore, we chose a combination of GSH and CALB in order to investigate the dual-responsiveness of the system. As controls we chose pure doxorubicin in PBS as well as DR-dPGS@DOX in PBS without any stimulus. The results from the release study can be seen in Figure 4b.

The experiment renders information about the on-going release during degradation. The free DOX was released completely within the first 8 h while the release of DR-dPGS@DOX in PBS occurred in a sustained manner. Furthermore, the dual-responsiveness of the polymer conjugate can be shown with reductive and/or enzymatic conditions. Incubation with 10 mM GSH renders a release of 72% of drug over time, enzymatic degradation with CALB leads to 79% drug release. Interestingly, a synergistic effect of the combination of both stimuli can be detected, with the combined conditions leading to 85% release of the loaded drug within 72 h.

### 3.5. Cytotoxicity and Cell Uptake

In order to investigate the effect of this novel copolymer on cells and to verify the cytotoxic behavior of drug-loaded carriers, we performed cytotoxicity studies using three different cancer cell lines: HeLa cervix carcinoma cells, A549 lung carcinoma cells and MCF-7 breast cancer cells (Figure S3). The experiments show that the copolymers DR-dPGS without the drug doxorubicin were well tolerated until the highest tested concentration of 1 mg mL^−1^. DOX encapsulated in DR-dPGS, as well as free doxorubicin, caused a similar concentration dependent decrease in the cells’ viability in all three cells lines. The effect of free and encapsulated doxorubicin was best in HeLa and A549 cells; MCF-7 cells seemed to be more robust against the drug. The results indicate that the cytotoxic properties of the drug are amplified by the copolymer, which can be further proven by the IC50 values obtained from these measurements and can be seen in Table 2. Interestingly, DR-DPGS@DOX outperforms the free drug by roughly an order of magnitude.

**Figure 5:**
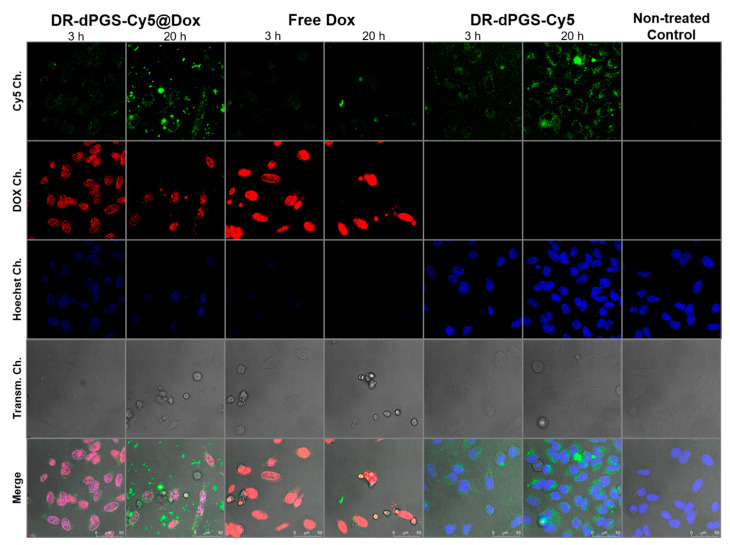
Confocal Microscope images of HeLa cells treated with DR-dPGS-Cy5 with and without DOX, free DOX for 3 h and 20 h, respectively, and nontreated cells. Blue: Hoechst (nuclei); red: DOX, green: Cy5-labelled carrier. Scale bar indicates 50 µm.

In order to visualize the cellular uptake and fate of the DR-dPGS and DOX, we used confocal laser scanning microscopy (CLSM). For this study we monitored living HeLa cells treated with DR-dPGS and DR-dPGS@DOX for 3 h and 20 h. DR-dPGS was labelled before imaging and loading with a hydrophobic cyanine dye (Cy5). The images displayed in Figure 5 clearly show that DR-dPGS and DOX are taken up by the cells and their signals accumulate over time. The DR-dPGS is mainly located in the cytosol outside the nucleus in distinct areas. These spots are most probably lysosomes and point to an endocytotic uptake pathway of DR-dPGS as shown for other sulfated polymers [35]. In contrast DOX is mainly located in the nucleus, which is in line with the literature and proves that the drug is released from DR-dPGS [36]. Furthermore, this can also be seen by the drastically reduced Hoechst signal, as DOX also interacts with DNA and thus replaces Hoechst [37]. Moreover, after 20 h of incubation the toxic effect of DOX is clearly seen as fewer cells can be observed and the remaining cells show morphological changes compared to nontreated cells or cells only treated with empty DR-dPGS. Therefore, it can be concluded that DR-dPGS can successfully transport and release doxorubicin in cancer cells.

## 4. Discussion

The copolymerization of glycidol, SSG and ε- caprolactone was investigated with regard to the impact of different catalysts on molecular weight, disulfide and ester content as well as degree of branching. After analysis by means of GPC, ^1^H and ^13^C NMR, IR and Raman spectroscopy, the Sr(OiPr)_2_-catalyzed polymer DR-dPG was selected and its degradation under reductive or enzymatic conditions could be proven by monitoring the signals of protons next to the disulfide and ester bond, respectively. The polymer was then further sulfated and DR-dPGS was loaded with the hydrophobic drug DOX. A DLC of 2.7% was calculated based on UV/Vis measurements. A release study with 10 mM GSH and CALB revealed that the drug can be released via reductive and enzymatic stimuli, as well as a synergistic effect of both, leading to 85% drug release over 72 h. Cytotoxicity measurements with three different cancer cell lines show that the polymer itself is well-tolerated, whereas DR-dPGS@DOX showed enhanced cytotoxic effects compared to the free drug, indicating that the cytotoxic behavior of the drug is maintained after encapsulation. This could also be shown with calculated IC50 values. The cellular uptake of dye-labeled DR-DPGS@DOX was examined with confocal laser scanning microscopy. Observations indicate that the carriers are taken up efficiently by HeLa cells. The polymer itself accumulates in the cytosol and releases the drug, which accumulates in the cell nucleus. These properties together with simple and gram scale synthesis make the synthesized copolymer a promising candidate for future biomedical applications.

## Figures and Tables

**Table 1:** Polymer synthesis and catalyst screening for polymerizations.

Catalyst	Mn ^(a)^ [kDa]	Mw ^(a)^ [kDa]	Đ	SS Content ^(b)^ [%]	SS Content ^(c)^ [%]	CL Content ^(b)^ [%]	DB ^(b)^ [%]	Yield [g]
Sn(Oct)_2_	12.8	17.1	1.3	2.3	2.6	5.8	27	1.2
Sr(OiPr)_2_	23.4	38.8	1.6	4.2	3.5	5.2	56	1.8
DBU	1.2	3.4	2.8	4.2	3.6	3.5	56	1.0
TBD	0.4	3.1	7.8	3.7	3.9	6.8	50	2.0
Mg(HMDS)_2_	<0.5		N/D	N/D	3.6	N/D	N/D	2.9
DPP	<0.5		N/D	N/D	N/D	N/D	N/D	0

(a) measured by GPC in DMF; (b) measured by ^1^H NMR; (c) measured by EA.

**Table 2:** Calculated IC50 values for DR-dPGS@DOX and free DOX.

	IC50 DR-dPGS@DOX (µg/mL)	IC50 DOX (µg/mL)
HeLa	0.036	0.117
A549	0.039	0.2549
MCF-7	0.444	6.163

## Data Availability

Data are contained within the article or Supplementary Materials.

## Supplementary Materials

The following are available online at https://www.mdpi.com/2073-4360/13/6/982/s1, Figure S1:1H NMR spectra obtained by the degradation study; Figure S2: DOX calibration; Figure S3: Cell viability results obtained from the cell viability assay (CCK-8) upon incubation of A549, HeLa and MCF-7 cells with (a) DR-dPGS, (b–d) DR-DPGS@DOX (DLC = 2.7%) and free DOX after 48 h of treatment; Figure S4: 1H NMR spectrum of the SSG monomer; Figure S5: 13C spectrum of the SSG monomer; Figure S6: 1H NMR spectrum of the Sn(Oct)2-catalyzed polymer; Figure S7: 13C NMR spectrum of the Sn(Oct)2-catalyzed polymer; Figure S8: 1H NMR spectrum of the Sr(OiPr)2-catalyzed polymer; Figure S9: 13C NMR spectrum of the Sr(OiPr)2-catalyzed polymer; Figure S10: 1H NMR spectrum of the DBU-catalyzed polymer; Figure S11: 13C NMR spectrum of the DBU-catalyzed polymer; Figure S12: 1H NMR spectrum of the TBD-catalyzed polymer; Figure S13: 13C NMR spectrum of the TBD-catalyzed polymer; Figure S14: GPC elugram of DR-dPG.
