# Supplementary material for: Gram Scale Synthesis of Dual-Responsive Dendritic Polyglycerol Sulfate as Drug Delivery System
Source: Polymers (Basel). 2021 Mar 23;13(6):982. doi: 10.3390/polym13060982 (PMC8004855; doi:10.3390/polym13060982)
Supplement: Supplementary file 1 [file polymers-13-00982-s001.pdf]

Supplementary Information

Gram Scale Synthesis of Dual-Responsive Dendritic Polyglycerol Sulfate as Drug Delivery System

Felix Reisbeck, Alexander Ozimkovski, Mariam Cherri, Mathias Dimde, Elisa Quaas, Ehsan Mohammadifar*, Katharina Achazi*, and Rainer Haag*

Institute of Chemistry and Biochemistry, Freie Universität Berlin, Takustr. 3, 14195 Berlin, Germany; freisbeck@zedat.fu-berlin.de (F.R.); saschaoz@zedat.fu-berlin.de (A.O.); mcherri6@zedat.fu-berlin.de (M.C.); mathias.dimde@fu-berlin.de (M.D.); equaas@zedat.fu-berlin.de (E.Q.);
Correspondence: haag@chemie.fu-berlin.de (R.H.); Tel.: +49-30-838-52633,
katharina.achazi@fu-berlin.de (K.A.) Tel.: +49-30-838-59145
ehsan@zedat.fu-berlin.de (E.M.) Tel.: +49-30-838-52632

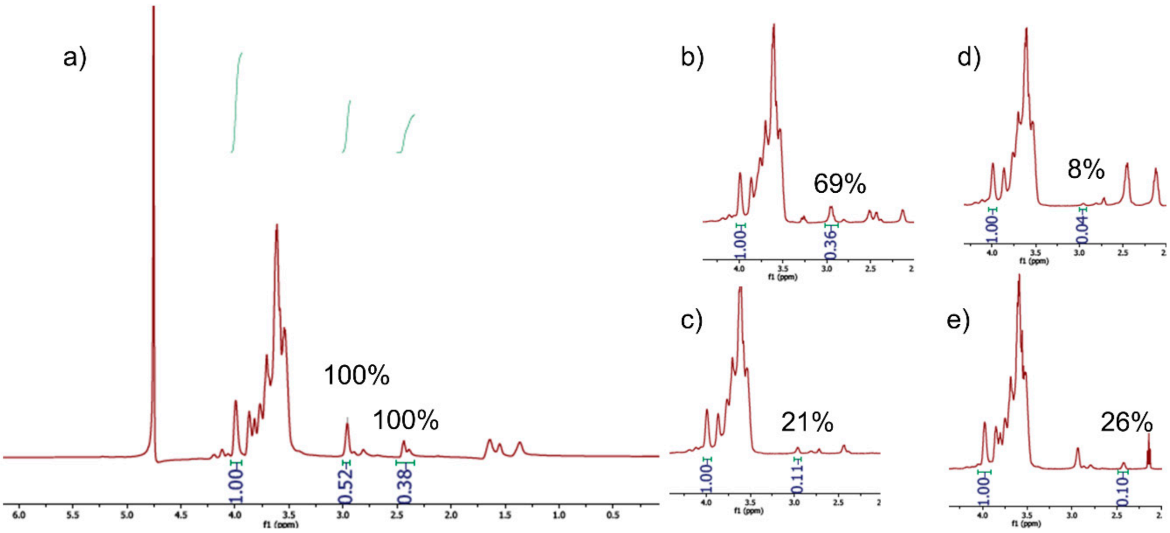

Figure S1: ^{1}H NMR spectra obtained by the degradation study. a) Original polymer without bond cleavage. Integrals of the disulfide-related and ester-related signals are considered 100%; b) Spectrum obtained with 10 mM GSH; c) Spectrum obtained with DTT; d) Spectrum obtained with TCEP; Spectrum obtained with CALB

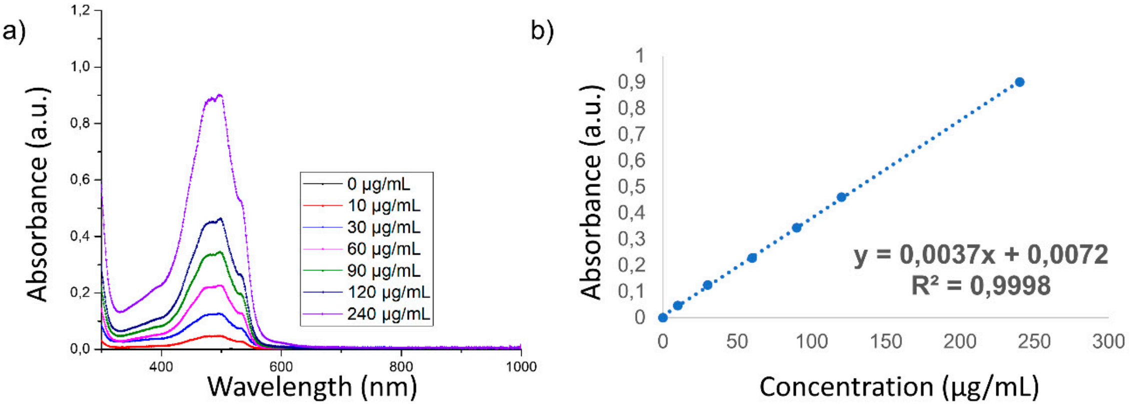

Figure S2: DOX calibration. a) UV/Vis measurements at different concentrations; b) Calibration curve obtained

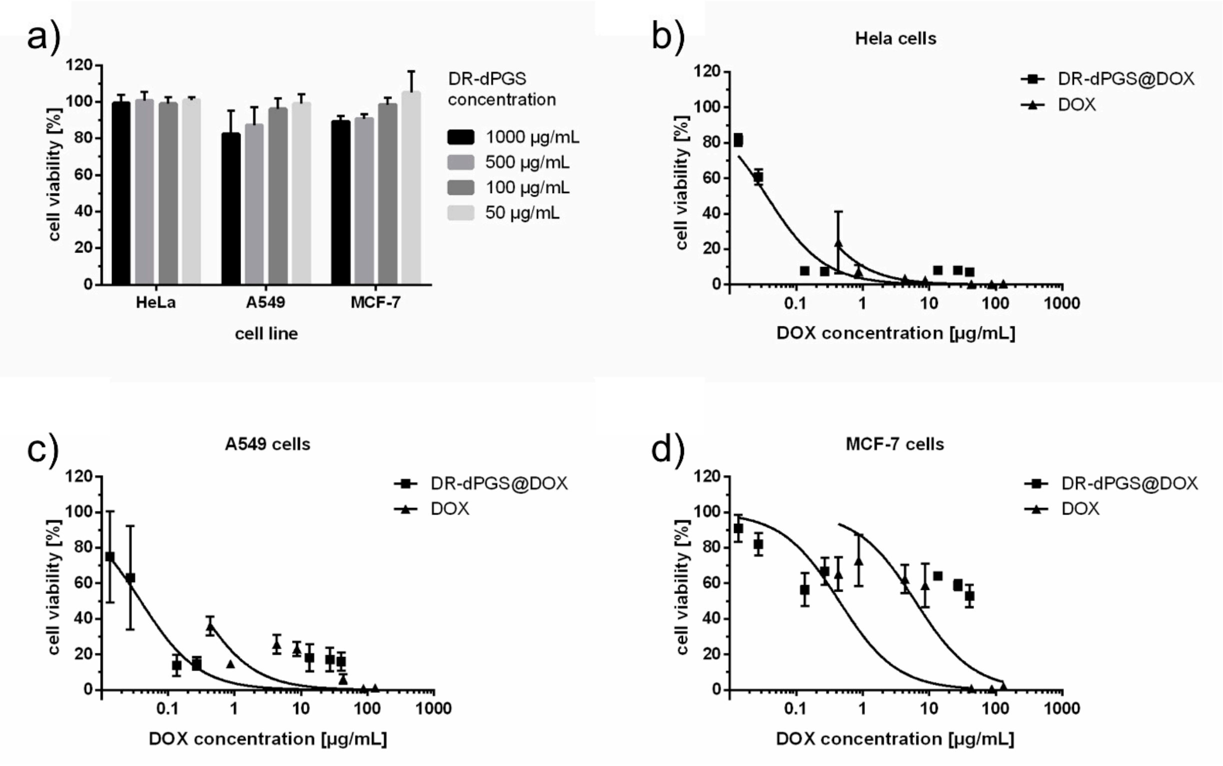

Figure S3: Cell viability results obtained from the cell viability assay (CCK-8) upon incubation of A549, HeLa cell and MCF-7 cells with a) DR-dPGS, b)-d) DR-dPGS@DOX (DLC = 2.7%) and free DOX after 48 h of treatment. The cell viability was normalized to 100% using the non-treated control. B)-D) To obtain the IC50 of encapsulated and free DOX, a log(inhibitor) vs. normalized response equation was used for fitting. Measurements were performed in triplicates and repeated three times. Data is represented as the mean of the three independent experiments with standard deviation.

Calculation of monomer content listed in Table 1:

The disulfide content based on ^1H NMR was calculated by the ratio of the integral of the dPG backbone (=∫ PG) to the methylene bridges (=∫ SS set to 4), subtracting the two protons corresponding to the caprolactone overlapping with the protons of the dPG backbone, as follows:

$$SS\% = \left(\frac{\int PG - \int CL}{5} + 1\right)^{-1} \times 100 = \left(\frac{\int PG - 2}{5} + 1\right)^{-1} \times 100 \qquad (S1)$$

The amount of ε-CL incorporated into the polymer was calculated in a similar approach, considering the ratio of ∫ PG to the integral of the methylene protons next to the carbonyl group (=∫ CL set to 2):

$$CL\% = \left(\frac{\int PG - \int CL}{5} + 1\right)^{-1} \times 100 = \left(\frac{\int PG - 2}{5} + 1\right)^{-1} \times 100 \qquad (S2)$$

Another way to determine the disulfide content is the measurement of elemental analysis. Here, as an apporiximation, the value for disulfide content (SS%) can be determined by relating the sulfur content measured in elemental analysis (SEA%) to the sulfur content of the original feed Sfeed% (Glycidol: 10, SSG: 1, ε-CL: 1 = 6.02), including the original molar ratio in the feed and dividing by two for the disulfides:

$$SS\% = \frac{S_{EA}\%}{S_{feed}\%} \times 0.1 \times 0.5 = \frac{S_{EA}\%}{6.02} \times 0.05 \qquad (S3)$$

Calculation of the degree of sulfation (DS):

The calculation for degree of sulfation was based on results obtained by 1H NMR. As stated in Table 1, DR-dPG has 4.2% disulfide and 5.2% ε-caprolactone incopoporated in its structure, respectively. The calculated elemental anaylsis obtained for 1000 sulfated glycidol molecules, 42 sulfated SSG molecules and 52 ε-CL molecules leads to the following distribution and was considered the theoretically maximal composition: C 22.12, H 3.13, Na 12.29, O 43.95 and S 18,52 %. The Sulfur content measured (14.08%) was then set in ratio to the theoretically possible amount and renders a degree of sulfation of 76%.

Calculation of the Mn of sulfated polymer:

For dPG, sulfation leads to roughly an increase of double the molecular weight, as statistically each glycidol unit leads to the formation of one hydroxyl group which can be converted into a sulfate group. Here, we first took the amount of converted hydroxyl groups into account:

$$N(OH) = \frac{DS \times Mn \times (\mathbf{100} - \boldsymbol{SS}\% - \boldsymbol{CL}\%)}{M(Glycidol)} = \frac{23400 \times 0.76 \times (1 - 0.042 - 0.052)}{74.08} \approx 217 \qquad (S4)$$

As hydroxyl groups are converted into sodium sulfates , the molecular weight of DRdPGS can be calculated with the following equation:

$$Mn(sulfated) = Mn - N(OH) + N(OH) \times M(SO_3^- Na^+) = [23400 - 217 + 217 \times 103.05]\frac{g}{mol} = 45545\ g/mol \qquad (S5)$$

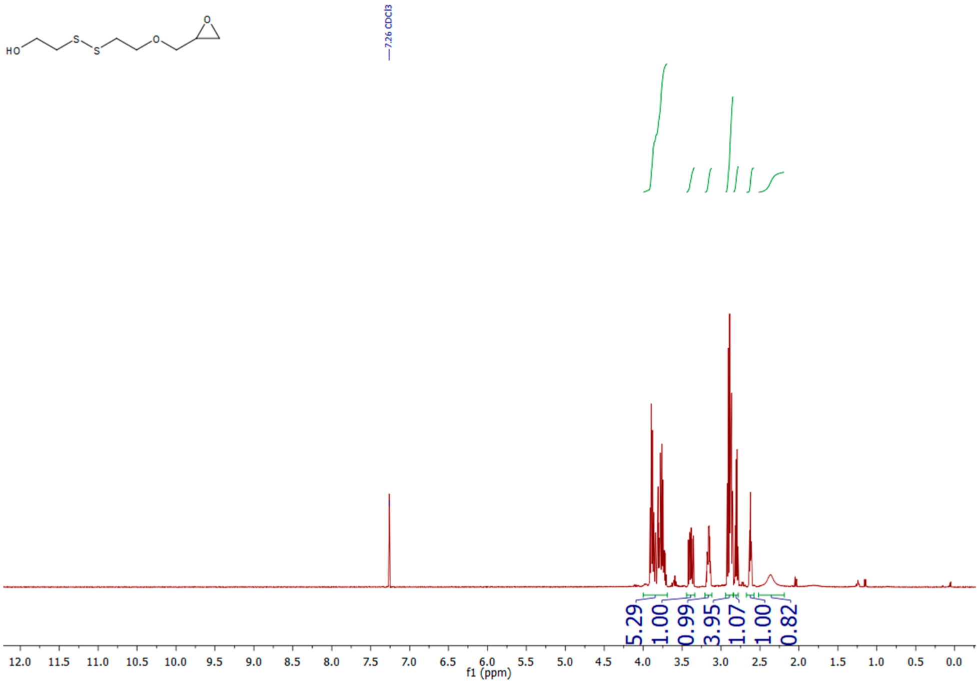

Figure S4: ^{1}H NMR spectrum of the SSG monomer

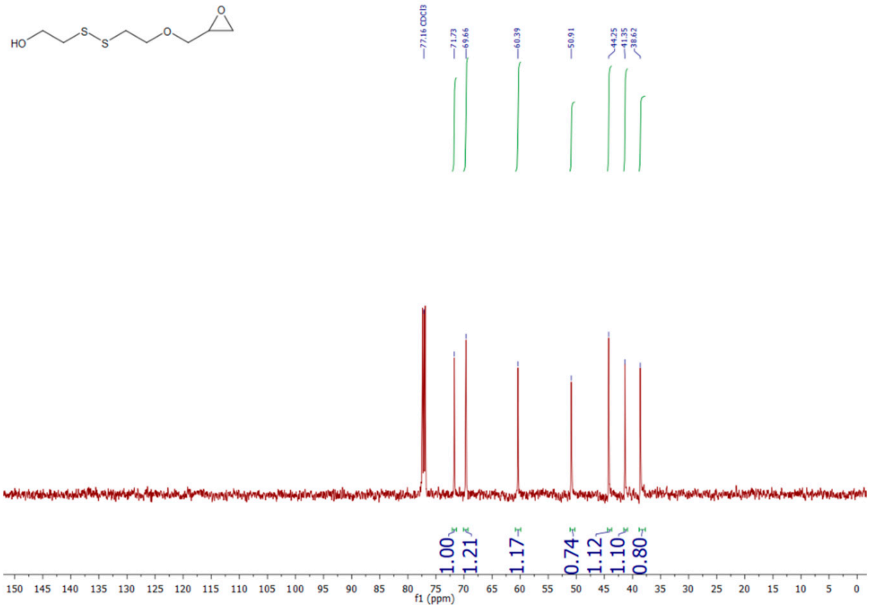

Figure S5: ^{13}C NMR spectrum of the SSG monomer

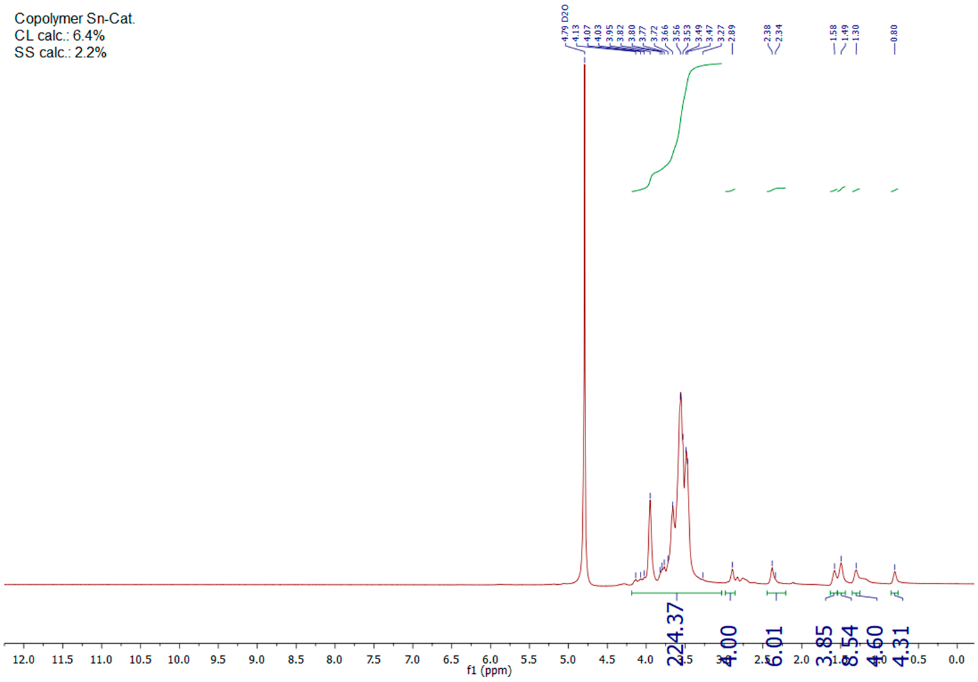

Figure S6: ¹H NMR spectrum of the Sn(Oct)$_2$-catalyzed polymer

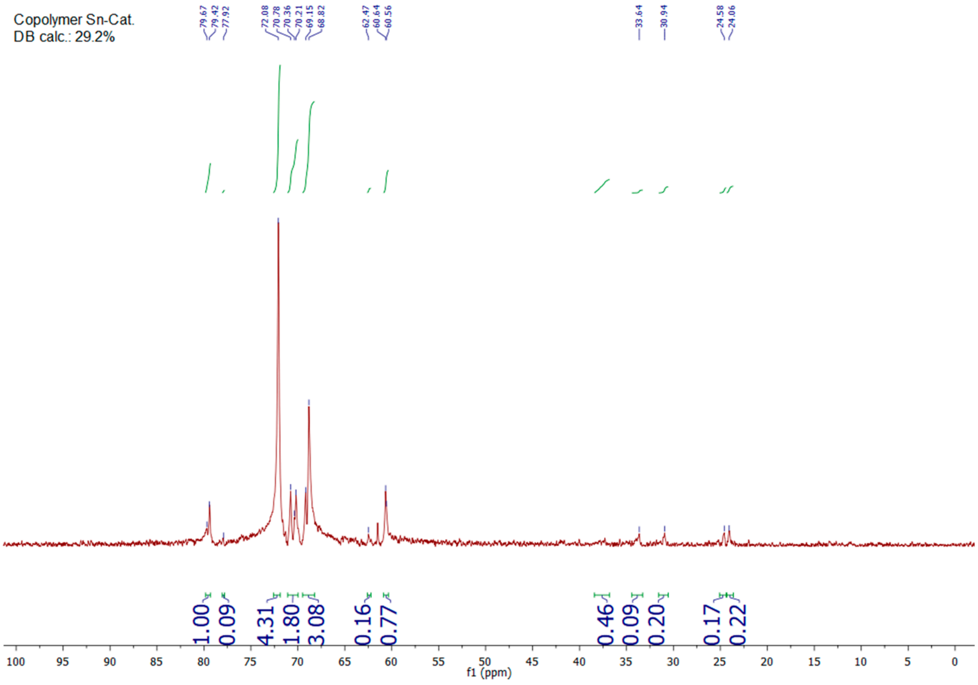

Figure S7: ¹³C NMR spectrum of the Sn(Oct)$_2$-catalyzed polymer

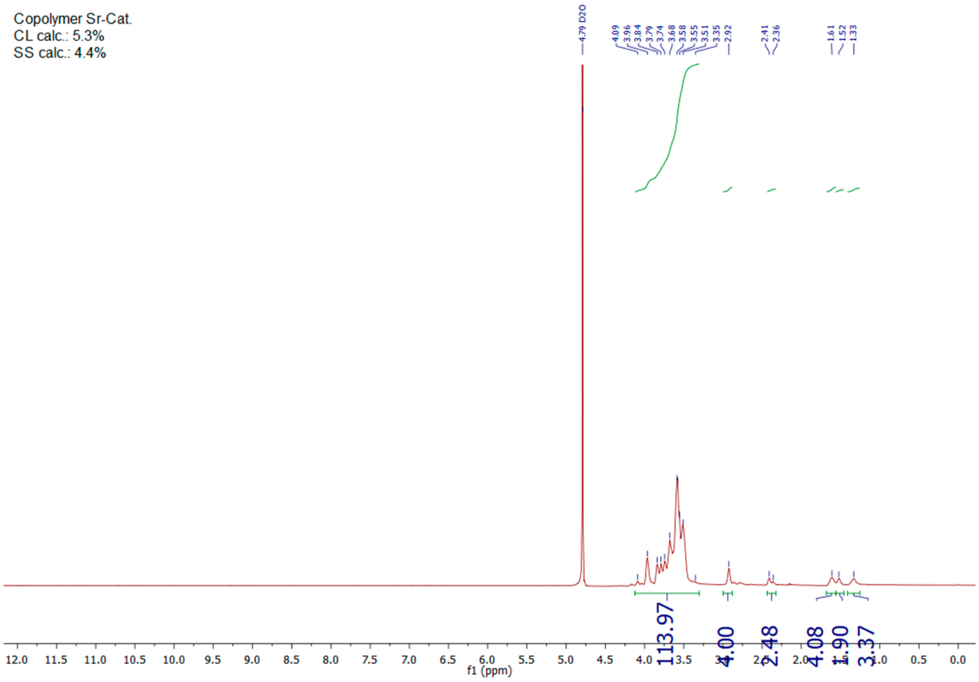

Figure S8: ^{1}H NMR spectrum of the Sr(OiPr)$_2$-catalyzed polymer

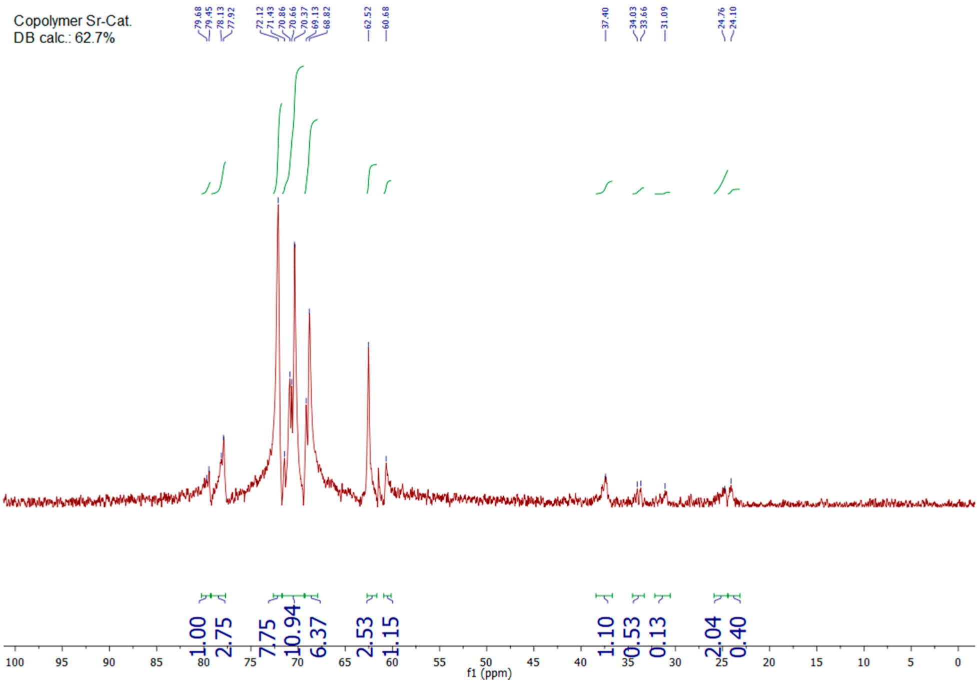

Figure S9: ^{13}C NMR spectrum of the Sr(OiPr)$_2$-catalyzed polymer

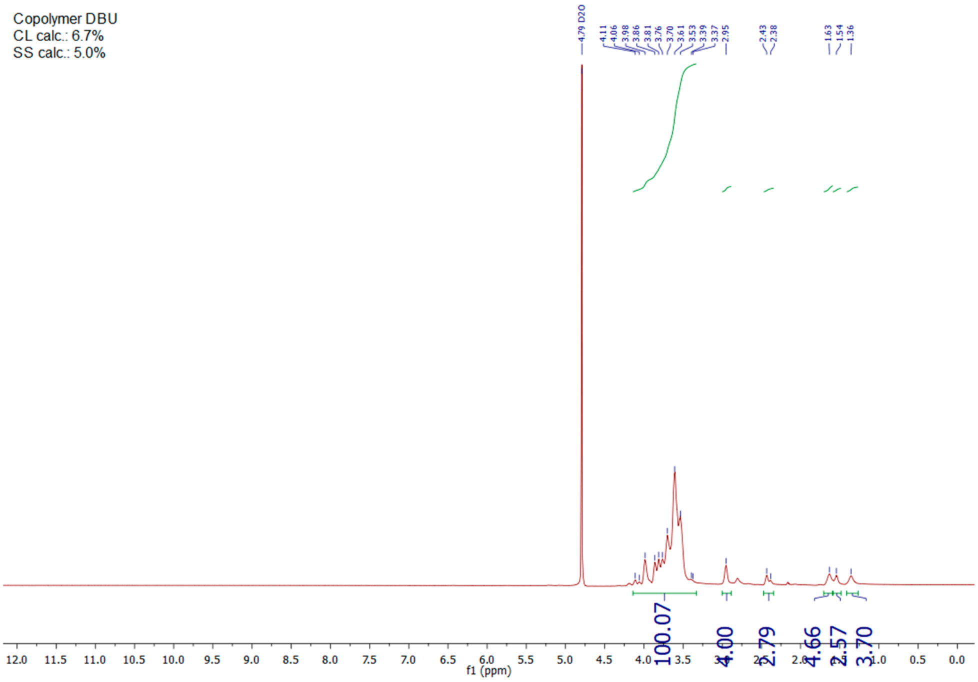

Figure S10: ^{1}H NMR spectrum of the DBU-catalyzed polymer

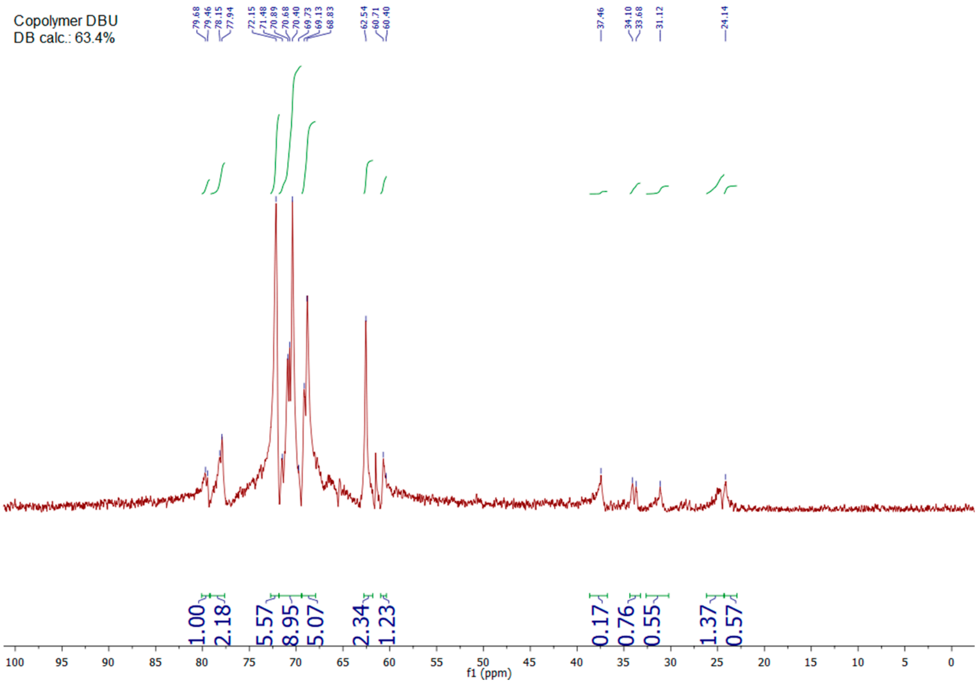

Figure S11: ^{13}C NMR spectrum of the DBU-catalyzed polymer

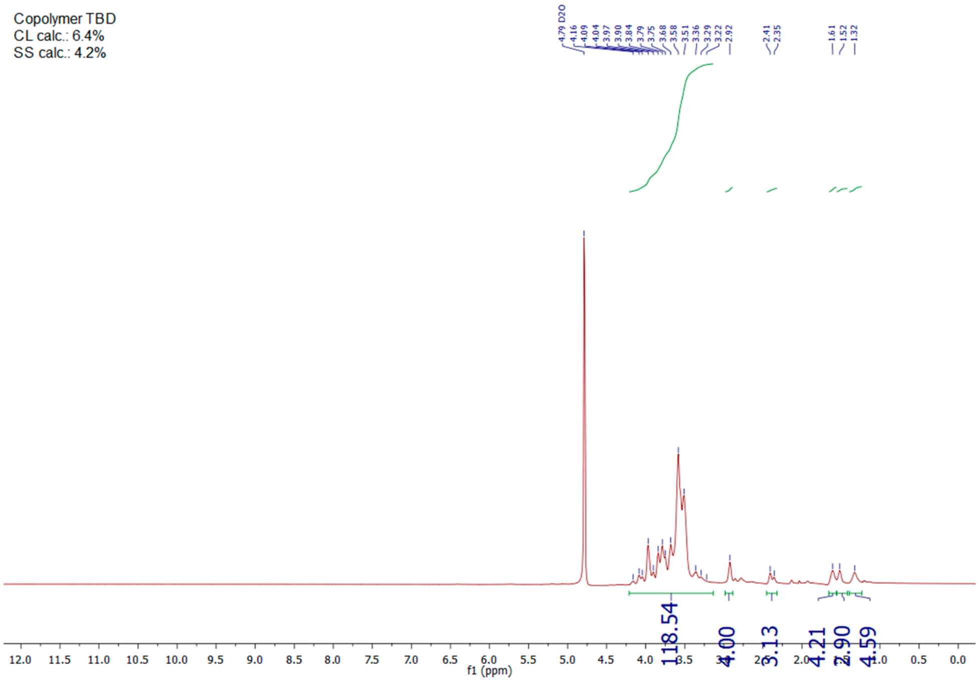

Figure S12: ^{1}H NMR spectrum of the TBD-catalyzed polymer

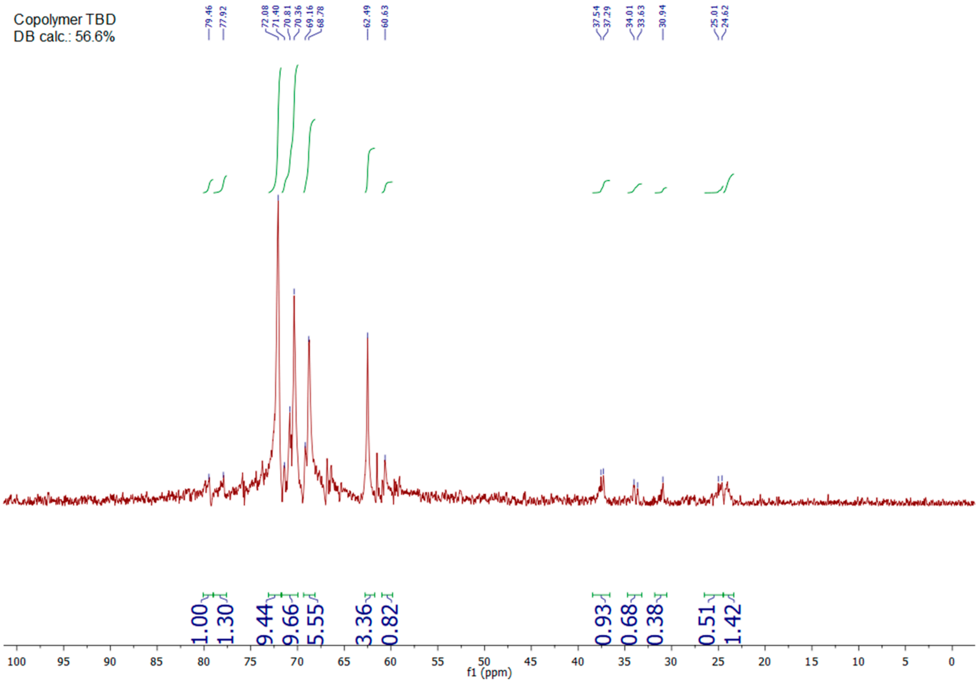

Figure S13: ^{13}C NMR spectrum of the TBD-catalyzed polymer

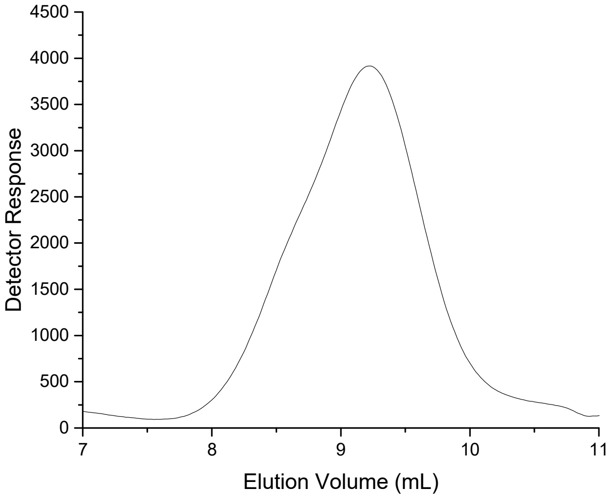

Figure S14: GPC elugram of DR-dPG
